# Supplementary material for: Eph and Ephrin Variants in Malaysian Neural Tube Defect Families
Source: Genes (Basel). 2022 May 26;13(6):952. doi: 10.3390/genes13060952 (PMC9222557; doi:10.3390/genes13060952)
Supplement: Supplementary file 1 [file genes-13-00952-s001.zip › genes-1694078-supplementary.pdf]

Table S1 List of human spina bifida genes in addition to lists that were reviewed in Greene et al., (2009) Hum Mol Genet 18(R2):p.R113-29 (40 genes) and Mohd-Zin et al., (2017) Scientifica (Cairo) 2017:p.5364827 (8 genes)

No.	Genes	Population and sample size of spina bifida studies showed association with genes related to NTDs or risk factor for spina bifida	References
1	*AMBRA1*	352 NTD cases and 224 controls	Ye *et al., (*2020) Hum Mutat 41(8):1393-93
2	**CELSR1/*SCRIB*	3 Spina bifida	Wang *et al.* (2018) Mol Genet Metab 124(1):94-100
3	*GIF*	14 spina bifida and 16 controls	Fofou-Caillierez *et al., (*2019) Am J Clin Nutr 109(3):674-83
4	*GLI2*	70 spina bifida and 52 controls (Chinese)	Lu *et al.,* (2016) Mol Neurobiol 53(8):5413-24
5	*GRHL3*	233 spina bifida	Lemay *et al., (*2017) Hum Mutat 38(6):716-24
6		503 NTD cases with total 757 probands including parents and 519 controls	Yang *et al., (*2019) Birth Defects Res 111 (19):1468-78
7	*GPR161*	6 spina bifida	Kim *et al., (*2019) Hum Mol Genet 28(2):200-208
8	*IRF6*	2 spina bifida	Kousa *et al., (*2019) Hum Mol Genet 28(10):1726-37
9	**MTRR*	2 Chinese family (exon 1)	Zhang *et al., (*2017) Neuromolecular Med 19(2-3):387-94
		7 NTD patients, 12 NTD parents and 5 controls	Tan *et al., (*2020) Neuroscience Research Notes 3(1):24-31
10	*PTK7*	192 spina bifida and 190 controls (California population)	Lei *et al.,* (2019) Mol Genet Genomic Med 7(4):e00584
11	*PTK7/*SCRIB*	1 spina bifida	Wang *et al., (*2018) Mol Genet Metab 124(1):94-100
12	*RAD9B* DNA damage response (DDR) gene	Found in 8 from 409 patients and none in 298 controls	Cao *et al., (*2020) Hum Mutat 41(4):786-99
13	**SARDH* (folate-mediated	270 NTD cases and 192 controls (Han Chinese)	Piao *et al., (*2016) Birth Defects Res A Clin Mol Teratol 106(4):232-39
14	*TNIP1*	2 families	Francesca *et al., (*2016) Childs Nerv Syst 32(6):1061-67
15	*WNT2B*	3 spina bifida (Chinese)	Bai *et al, (*2016) Mol Med Rep 13(1):99-106
16	*WNT7B*		

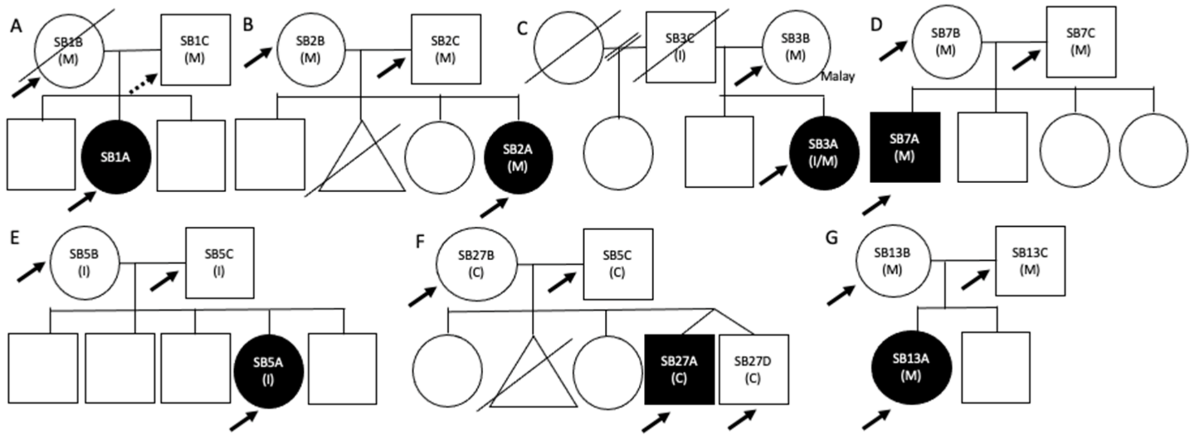

Figure S1. Pedigree of the 7 unrelated individuals with sporadic spina bifida and their unaffected family members, where available. (A, B, D, E, G) 5 complete trios with mother-father-proband. (F) 1 triad-family with mother-father-proband-twin sibling. (C) 1 single-parent family with mother-proband (SB= spina bifida family; number= family ID; A= proband; B= mother; C= father; D= sibling; circle= female; square= male; black shape= affected with spina bifida; 1 slash line= deceased; 2 slash lines= diseased spoused; triangle= unknown gender; horizontal line= marriage; vertical line= children; diagonal line= twin; arrow with straight line= DNA sample and exome data set available; arrow with dashed line= DNA sample available but exome data set not available; M= Malay ethnicity; I= Indian ethnicity; C= Chinese ethnicity; slash= mixed ethnicity)

Table S2 Clinical features of the 7 probands sent for whole exome sequencing

Spina bifida probands	Clinical features
Spina bifida occulta (SB1A, 2A, and 3A)	Spina bifida occulta (SBO) of the severe neurological deficit type. Dysfunctional ambulation present. Hip dislocation present in SB2A, and 3A. Bladder and bowel deficits inclusive in all 3 probands.
Spina bifida occulta (SB7A)	SBO of the less severe neurological deficit type. Able to ambulate and walk but with bladder and bowel impairment. As well as growing weakness in left leg.
Spina bifida aperta (SB5A)	Spina bifida aperta (SBA) with open lesion and ventriculo-peritoneal (VP) shunt
Syndromic spina bifida aperta (SB27A)	SBA with open lesion, VP shunt, and craniosynostosis
Syndromic spina bifida aperta (SB13A)	SBA with open lesion, VP shunt and Turner Syndrome

Table S3 Primers used in this study for screening and validation of the human variants

No.	Genes	rs number	Accession ID (GRCh37.p13; Hg19)	Primers	Expected size (bp)
1	*EPHA2*	rs147977279	NC_000001.10	Forward: 5′ GGGGCATCTTTAACCCCTTC 3′<br>Reverse: 5′ TCTCGCTCTGTCTGAGAGTT 3′	537
2	*EPHB6*	rs780569137	NC_000007.13	Forward: 5′ AACTGAACGTCAAAGAGCGG 3′<br>Reverse: 5′ GTGCAAGACGAGTGGGC 3′	392
3	*EFNB1*	rs772228172	NC_000023.10	Forward: 5′ TCTTCCAAGGGACAGCGAT 3′<br>Reverse: 5′ CACTCGAAAAAGCCGGAGA 3′	692

Table S4 *Eph* and *ephrin* variants screened in 7 spina bifida probands (Proband SB1A to Father of SB7A)

Gene	rs number	MAF	Proband SB1A	Mother of SB1A	Proband SB2A	Mother of SB2A	Father of SB2A	Proband SB3A	Mother of SB3B	Proband SB7A	Mother of SB7A	Father of SB7A
EPHA2	rs374825051	**0**	**Het**	**Het**	Wt	Wt	Wt	Wt	Wt	Wt	Wt	Wt
EPHA2	rs147977279	**0**	Wt	Wt	**Het**	Wt	**Het**	Wt	Wt	Wt	Wt	Wt
EPHA3	rs17801309	0.0549	Wt	Wt	Wt	Wt	Wt	Wt	Wt	Wt	Wt	Wt
EPHA5	rs36050417	0.021	Wt	Wt	Wt	Wt	Wt	Wt	Wt	Wt	Wt	Wt
EPHA7	rs2278106	0.064896	Wt	Wt	Wt	Wt	Wt	Wt	Wt	Wt	Wt	Wt
EPHA7	rs2278107	0.065	Wt	Wt	Wt	Wt	Wt	Wt	Wt	Wt	Wt	Wt
EPHA8	rs76452513	0.09	Wt	Wt	Wt	Wt	Wt	Wt	Wt	**Het**	Wt	**Het**
EPHA10	rs78757428	0.02	Wt	Wt	Wt	Wt	Wt	Wt	Wt	**Het**	Wt	**Het**
EPHB6	Chr07:142,562,076	**0**	Wt	Wt	Wt	Wt	Wt	**Het**	Wt	Wt	Wt	**Het**
EPHB6	rs8177146	0.05	Wt	Wt	Wt	Wt	Wt	**Het**	Wt	Wt	Wt	**Het**
EPHB6	rs780569137	**0**	Wt	Wt	Wt	Wt	Wt	Wt	Wt	Wt	Wt	Wt
EFNA3	rs17723260	0.08	Wt	Wt	Wt	Wt	Wt	Wt	Wt	**Het**	**Het**	Wt
EFNB1	rs772228172	**0**	**Het**	Wt	Wt	Wt	Wt	Wt	Wt	Wt	Wt	Wt

Table S4 Continued (Proband SB5A to Father of SB13A)

Gene	rs number	MAF	Proband SB5A	Mother of SB5A	Father of SB5A	Proband SB27A	Mother of SB27A	Father of SB27A	Twin-sibling of SB27D	Proband SB13A	Mother of SB13A	Father of SB13A
EPHA2	rs374825051	**0**	Wt	Wt	Wt	Wt	Wt	Wt	Wt	Wt	Wt	Wt
EPHA2	rs147977279	**0**	Wt	Wt	Wt	Wt	Wt	Wt	Wt	Wt	Wt	Wt
EPHA3	rs17801309	0.0549	Wt	Wt	Wt	**Het**	Wt	**Het**	Wt	**Het**	Wt	**Het**
EPHA5	rs36050417	0.021	Wt	Wt	Wt	**Het**	Wt	**Het**	Wt	Wt	Wt	Wt
EPHA7	rs2278106	0.064896	Wt	Wt	Wt	**Het**	**Het**	**Het**	**Het**	Wt	Wt	Wt
EPHA7	rs2278107	0.065	Wt	Wt	Wt	**Het**	**Het**	**Het**	**Het**	Wt	Wt	Wt
EPHA8	rs76452513	0.09	**Het**	**Het**	Wt	Wt	Wt	Wt	Wt	**Het**	Wt	**Het**
EPHA10	rs78757428	0.02	Wt	Wt	Wt	Wt	Wt	Wt	Wt	Wt	Wt	Wt
EPHB6	Chr07:142,562,076	**0**	Wt	**Het**	Wt	Wt	**Het**	Wt	Wt	Wt	Wt	Wt
EPHB6	rs8177146[#]	0.05	Wt	Wt	Wt	Wt	**Het**	Wt	**Het**	Wt	Wt	Wt
EPHB6	rs780569137	**0**	**Het**	**Het**	Wt	Wt	Wt	Wt	Wt	Wt	Wt	Wt
EFNA3	rs17723260	0.08	Wt	Wt	Wt	Wt	Wt	Wt	Wt	**Het**	**Het**	**Het**
EFNB1	rs772228172	**0**	Wt	Wt	Wt	Wt	Wt	Wt	Wt	Wt	Wt	Wt

Table S5 Minor allele frequency (MAF) and protein function prediction (PolyPhen2 HumDiv, PolyPhen 2 HumVar, SIFT, PROVEAN and CADD) of thirty selected variants of reported spina bifida-related genes

Reported spina bifida genes (Sorted by alphabet)	MAF (GnomAD when available)	Protein function prediction		
		Polyphen2 HumDiv & Polyphen2 HumVar (**PD1, PD2**, B)	SIFT (**D**,T) & Provean (**D**,N)	CADD (**T1%,** T10%, B90%)
1. *ALDH1A2:rs4646626:C>T:chr15:58256127:SB5A*	T=0.441161 (N= 250,872 alleles) (GnomAD_exomes)	B,B	T,N	T10%
**2. *ALDH1L1:rs2886059:C>A:chr03:125865766:SB2A***	A=0.159075 (N= 249, 486 alleles) (GnomAD_exomes)	B,B	**D,D**	T10%
3. *APEX1*:rs1130409: chr14:20,925,154:T>G:SB5A	G=0.433866/60763 (GnomAD)	B,B	T,N	B90%
**4. *BHMT*:rs3733890:chr05:78421959:G>A:SB2A**	A=0.302334 (N= 251,278 alleles) (GnomAD_exomes)	B,B	T,N	**T1%**
5. COMT:rs4680:G>A:chr22:19951271:SB1A	A=0.461091 (N= 247,346 alleles) (GnomAD_exomes)	B,B	T,N	T10%
**6. *CUBN:rs143400113:C>T :chr10:16975121:CUBN.1:SB1A***	**T=0.00076/191 (GnomAD_exomes)**	B,B	T,N	B90%
**7. *CUBN:rs1801224:G>T :chr10:17147521:CUBN.2:SB1A***	G=0.420811/58858 (GnomAD)	**PD2,**B	**D,D**	T10%
8. CUBN:rs1801231:G>A :chr10:17024503:CUBN.6:SB1A:SB2A	A=0.770228 (N=251,288 alleles) (GnomAD_exomes)	B,B	T,N	T10%
9. *CUBN:rs3740168:G>C :chr10:16948390:CUBN.3:SB1A*	C=0.044715 (11231/251168, GnomAD_exome)	B,B	T,N	T10%
**10. *CUBN:rs2271460:A>C :chr10:16961995:CUBN.4:SB1A***	C=0.017247/4335 (GnomAD_exomes)	**PD1, PD1**	**D,D**	**T1%**
**11. *CUBN:rs369981313:G>T :chr10:17110139:CUBN.5:SB1A***	**T=0.000264/37 (GnomAD)**<br>**T=0.0002/1 (1000Genomes)**	B,B	T,N	B90%
**12. *CUBN:rs62619939:C>G:chr10:16967586:CUBN.7:SB2A***	G=0.129916/32624 (GnomAD_exomes)	**PD1, PD1**	**D,D**	**T1%**
13. *GRHL3*:rs2486668 :chr01:24658063:SB2A:C>G	G= 0.157941 (N= 251,202 alleles) (GnomAD_exomes)	B,B	T,N	T10%
**14. *MTHFD1*:rs2236225:G>A:chr14:64908845:SB2A**	A=0.443848/111611 (GnomAD_exomes)	B,B	T,N	**T1%**
**15. *MTHFR:rs200947520:chr01:11850776:G>T:MTHFR.2:SB5A***	**T=0.000453/114 (GnomAD_exomes)**	B,B	T,N	T10%
**16. *MTHFR*:rs1801133 :chr01:11856378:G>A:MTHFR.1:SB5A**	A=0.314859/79177 (GnomAD_exomes)	**PD1, PD2**	**D,D**	**T1%**
17. *MTRR:rs10380:C>T:chr05:7897191:MTRR.3:SB2A*	T=0.156812/39347 (GnomAD_exomes)	B,B	T,N	T10%
18. *MTRR:rs162036:A>G:chr05:7885959:MTRR.2:SB2A*	G=0.175948/44240 (GnomAD_exomes)	B,B	T,N	T10%
**19. *MTRR: rs1801394:A>G :chr05:7870973:MTRR.1:SB1A:SB5A***	G=0.465808/117132 (GnomAD_exomes)	**PD1, PD1**	**D,**N	**T1%**
**20. *MTRR*:rs2287780:C>T :chr05:7889304:MTRR.4:SB1A**	T=0.061343/15425 (GnomAD_exomes)	**PD1, PD1**	**D,D**	**T1%**
**21. *PARD3*:rs118153230 :chr10:34630570:SB2A:C>T**	**T= 0.006427 (N= 248,708 alleles) (GnomAD_exomes)**	B,B	T,N	B90%
22. *PCMT1*:rs4816 :chr06:150114745:SB5A:G>A	A=0.464217/99400 (GnomAD_exomes)	B,B	T,N	T10%
**23. *PTCH1*:rs357564 :chr09:98209594:G>A:SB1A**	A= 0.325664 (N= 264,690 alleles) (TOPMED)<br>A= 0.3968 (N= 5,008 alleles) (1000Genomes)	**PD2,** B	**D,**N	**T1%**

Table S5 Continued

Reported spina bifida genes (Sorted by alphabet)	MAF (GnomAD when available)	Protein function prediction		
		Polyphen2 HumDiv & Polyphen HumVar (**PD1, PD2**, B)	SIFT (**D**,T) &PROVEAN (**D**,N)	CADD (**T1%,** T10%, B90%)
24. SCRIB:rs781978489:chr08:144886022:G>A:SB5A	**A=0.00004/9 (GnomAD_exomes)**	**PD1, PD1**	**D,D**	**T1%**
25. SOD2:rs4880:A>G:chr06:160113872:SB2A	G=0.483949/117709 (GnomAD_exomes)	B,B	T,N	T10%
26. TRDMT1:rs11254413:G>A:chr10:17204187:SB1A	A=0.113207/28254 (GnomAD_exomes)	B,B	T,N	B90%
**27. *TNIP1*:rs2233311:C>A :chr05:150410219:SB2A**	A=0.410176/101539 (GnomAD_exomes)	**PD1,** B	T,N	T10%
28. VANGL1:chr01:116206438:C>A:VANGL1.1:SB2A:SB5A	**A= Not reported**	**PD1, PD1**	T,N	**T1%**
29. VANGL1:rs4839469:G>A:chr01:116206423:VANGL1.2:SB1A	A=0.150493/37847 (GnomAD_exomes)	B.B	T,N	T10%
***30. XPD:ERCC2*:rs1799793:chr19:45,867,259:C>T:SB5A**	T=0.263102/36860 (GnomAD)	B,B	T,**D**	**T1%**

PolyPhen-2 (V2.2.3r406) prediction HumDiv and HumVar: **Probably Damaging (PD1), Possibly Damaging (PD2),** Benign (B); SIFT prediction (cut-off 0.05): **Damaging (D),** Tolerate (T); PROVEAN (cut-off -2.5): **Deleterious (D),** Neutral (N); CADD (CADD model GRCh37-v.16): **Top 1% (T1%)= >20 PHRED score,** Top 10% (T10%)= 10-20 PHRED score, Bottom 90% (B90%)= 0-10 PHRED score.
